# Supplementary material for: The effect of different structural decoration geometries on vibration propagation in spider orb webs
Source: PLoS One. 2025 Oct 29;20(10):e0332593. doi: 10.1371/journal.pone.0332593 (PMC12571255; doi:10.1371/journal.pone.0332593)
Supplement: S1 File — (DOCX) [file pone.0332593.s001.docx]

**Supplementary information**

**The effect of different structural decoration geometries on vibration propagation in spider orb webs**

G. Greco^1*$^, V. F. Dal Poggetto^2$^, L. Lenzini^&$^, F. Castellucci^3,4^, N. M. Pugno^2,5*^

^1^ Department of Anatomy Physiology and Biochemistry Swedish University of Agricultural Sciences Uppsala 75007, Sweden

^2^ Laboratory for Bio-Inspired, Bionic, Nano, Meta, Materials & Mechanics, Department of Civil, Environmental and Mechanical Engineering, University of Trento, Via Mesiano, 77, 38123 Trento, Italy

^3^ Department of Biological, Geological and Environmental Sciences—University of Bologna, via Selmi 3, 40126, Bologna, Italy

^4^ Zoology Section, Natural History Museum of Denmark—University of Copenhagen, Universitetsparken 15, 2100, Copenhagen, Denmark

^5^ School of Engineering and Materials Science, Queen Mary University of London, Mile End Road, London E1 4NS, UK

^&^ independent researcher

^$^ these authors contributed equally

*corresponding authors: [gabriele.greco@slu.se](mailto:gabriele.greco@slu.se) ; [nicola.pugno@unitn.it](mailto:nicola.pugno@unitn.it)


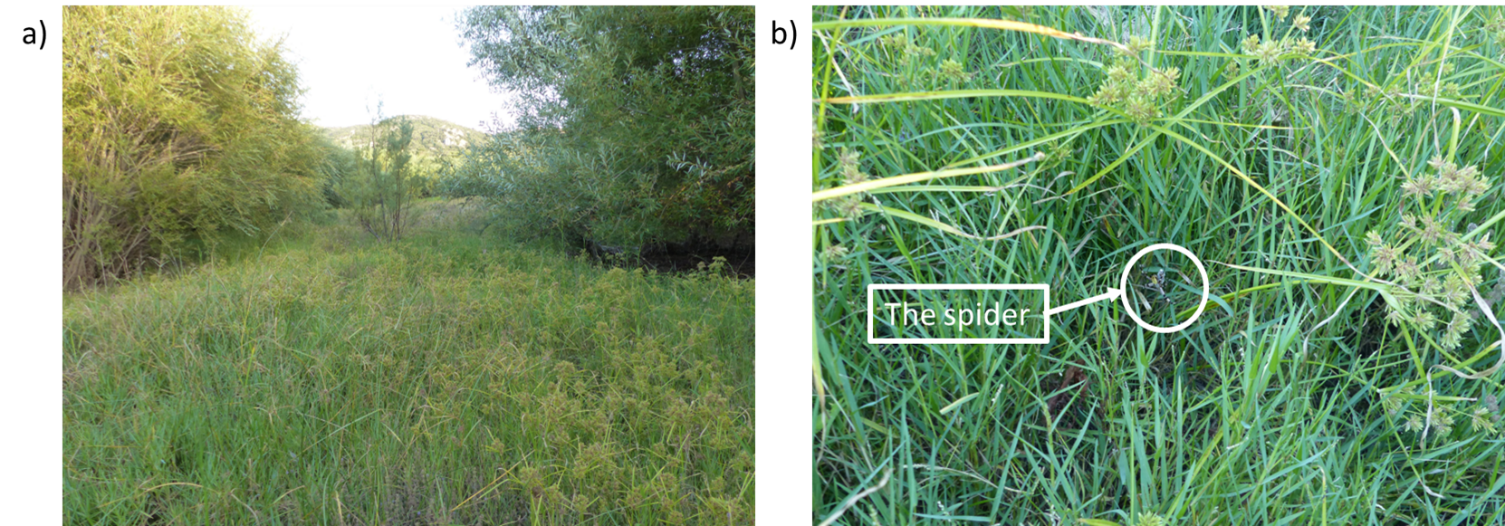


Figure S1: a, b) Typical habitat of Argiope bruennichi. The localities from which the stabilimentum sampled were: Li lucianeddi, Liscia river, and Saint Pasquale (Sardinia, Italy).

**
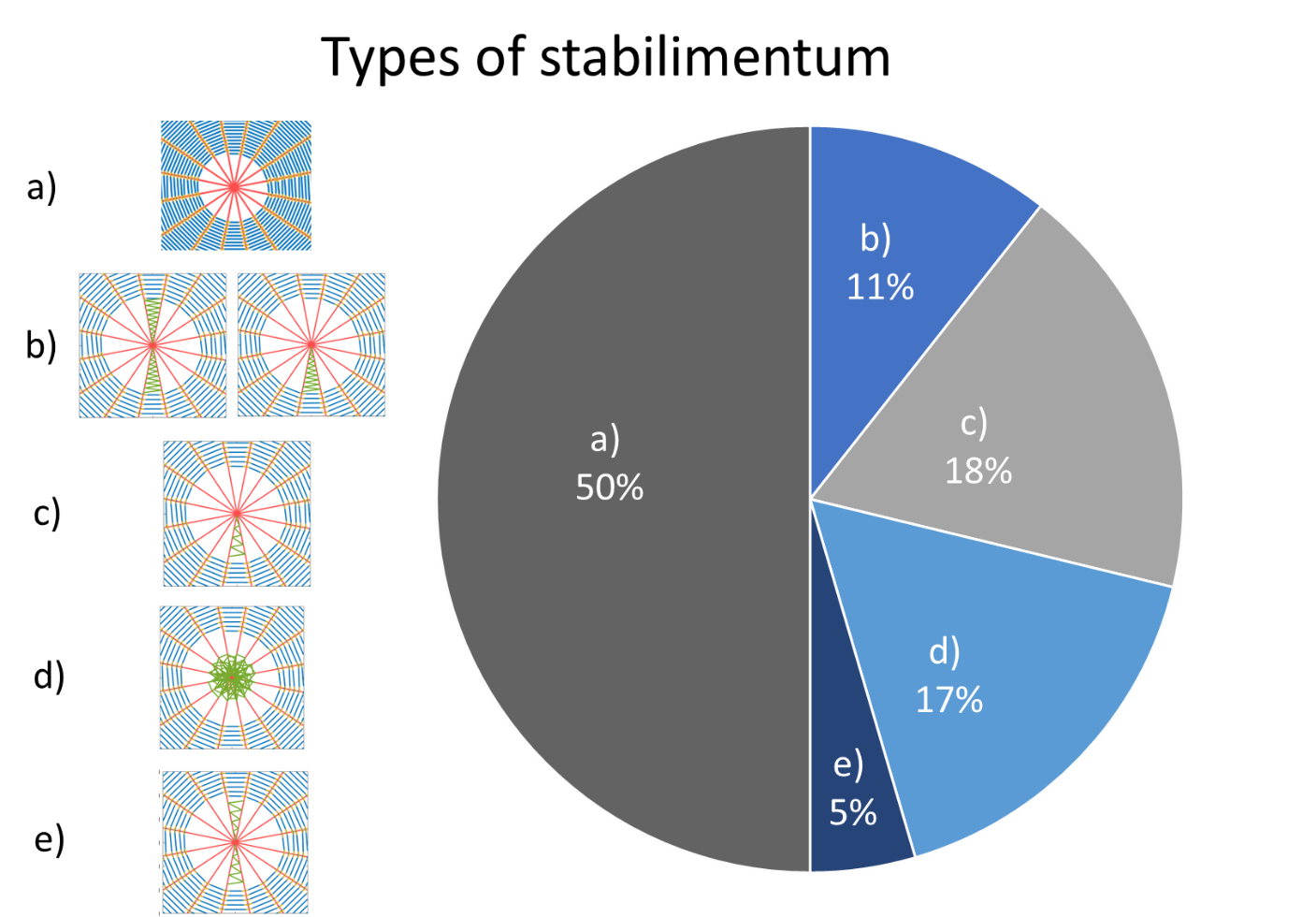
**

*Figure S2: Different types of stabilimentum occurrences observed in this study in the population of interest. a) Absent, b) normal and reduced, c) drafted, d) platform, and e) juvenile.*

Table S1: the observations of the stabilimenta in the region of interest.

| **Year** | **Locality** | **District** | **Province** | **Normal or reduced** | **Drafted** | **Platform** | **Juvenile** | **Absent** | **Total** |
| --- | --- | --- | --- | --- | --- | --- | --- | --- | --- |
| 2018 | Liscia river | Santa Teresa Gallura | OT | 9 | 23 | 11 | 0 | 69 | 112 |
| 2019 | S. Pasquale | Santa Teresa Gallura | OT | 1 |  | 1 | 6 | 1 | 9 |
| 2019 | Liscia river | Santa Teresa Gallura | OT | 6 | 3 | 5 |  | 20 | 34 |
| 2020 | S. Pasquale | Santa Teresa Gallura | OT |  |  |  | 3 | 4 | 7 |
| 2020 | Liscia river | Santa Teresa Gallura | OT | 8 | 19 | 25 | 3 | 35 | 90 |
| 2020 | Li Lucianeddi | Santa Teresa Gallura | OT | 4 | 3 | 2 |  | 3 | 12 |
| **Total** |  |  |  | 28 | 48 | 44 | 12 | 132 | 264 |

Supplementary section: Numerical simulations

The numerical model of the spider orb web is implemented using the finite element (FE) method [1], which allows the computation of the displacements of the structure at defined locations (nodes) and the derivation of the related quantities (e.g., strains and stresses). Nodes are used to discretize the structure and are connected by elements that behave according to assigned material properties. Figure SS1a shows the three types of materials considered: radial threads (red), spiral threads (blue), and gluing elements (yellow). Each of the sixteen radial threads, equally spaced around the origin of the Cartesian coordinate system (2π/16 angular spacing), has a length of 300 mm, discretized using 5 mm elements. Spiral threads are initially distributed following an Archimedean spiral starting from the distance of 100 mm from the system origin until reaching the distance of 300 mm, discretized using 5 mm elements. Gluing elements are used to connect the nodes that form radial and spiral threads and have a distance smaller than 10 mm. Figure S3b shows the considered stress-strain curves of the radial and (major ampullate, M.A., in red) spiral threads (flagelliform, in blue); gluing elements are considered with the same properties as spiral threads; elements used to model the stabilimentum are considered as aciniform threads (green). The equilibrium state of the orb web is reached by (i) connecting all radial threads with a prescribed pre-stress value of 50 MPa, (ii) overlaying spiral threads with an initial pre-stress value of 10 MPa, (iii) connecting nodes of radial and spiral threads using gluing elements, and (iv) applying a stabilization procedure to determine the final state of stresses which accommodates the structure in equilibrium (using a Newton-Raphson iterative scheme with non-linear material properties [2]). The achieved state of stress of the spider orb web is shown in Figure S3c.

*
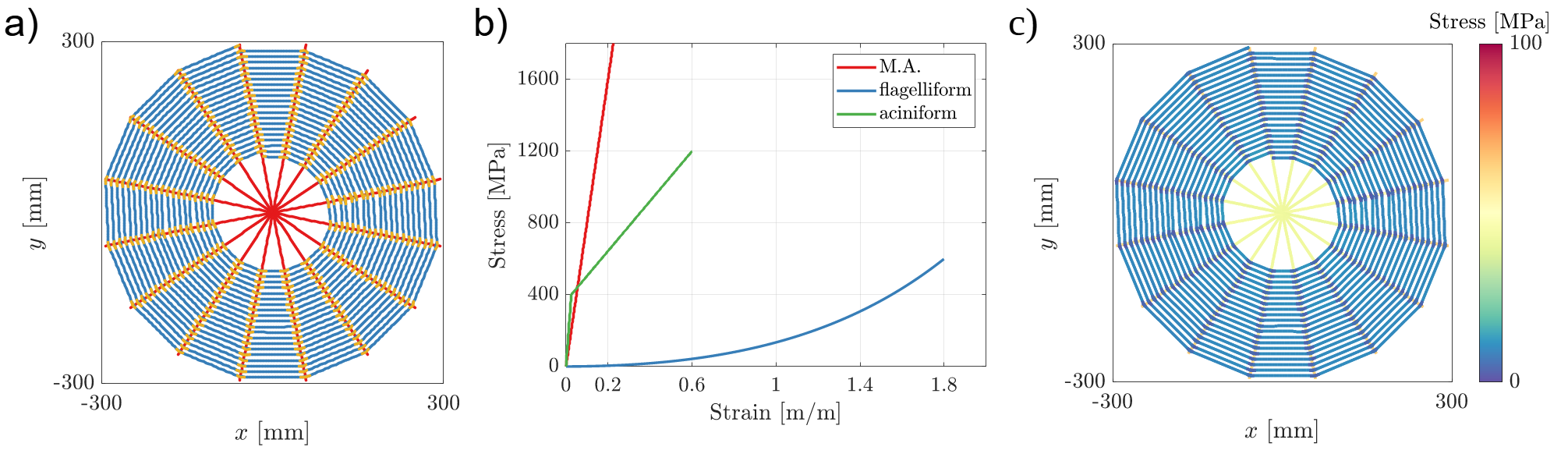
**Figure S3: Initial structure and applied pre-stresses at threads. (a) The initial structure is composed of radial threads (red), spiral threads (blue), and gluing elements (yellow) connecting radial and spiral threads. (b) Considered stress-strain curves of major ampullate (M.A., red), relative do radial threads, flagelliform (blue), relative to spiral threads, and aciniform (green), relative to stabilimentum elements (not shown, see Figure 1c for reference). (c) Stress state after stabilization process with 50 MPa stresses at radial threads and 10 MPa at spiral threads.*

The modelling of stabilimenta for inclusion follows those presented in Figure 1, connecting adjacent radial threads in various patterns. Most of the stabilimenta configurations considered here consist of winding patterns in adjacent radial threads. The stabilimenta indicated by Figures 1c (cases N, J, R, D) show winding patterns considering opposite pairs of radial threads with varying density. For the P case, nodes of consecutive radial threads are chosen randomly to create a non-trivial pattern. Each stabilimentum configuration is labelled according to the letters presented in Figure 1c.

*Time delays for the case of transverse vibrations*

As the time intervals necessary to achieve the displacement threshold are barely distinguishable when comparing the polar plots for the transverse vibration case, we report here the differences in time between the cases without and with stabilimentum, which are in the order of microseconds. The results for the transverse vibration direction are summarized in Figure S4, depicting the difference in time for different stabilimentum types (N, J, R, P, D) and input points (I_T_, I_TR_, I_R_, I_BR_, and I_B_), using polar plots. In each plot, the radial coordinate represents the time delay required to reach the displacement threshold (in this case, 3.75 µm) relative to the baseline. Differences are observed in the time delay as the amount of the stabilimentum structure increases across the different web structures. In the normal case (N, top central panel of Figure S4), the response delay exceeds 15 µs at output points O_4_/O_5_ and O_12_/O_13_, when prey impacts occur at I_T_ and I_B_, respectively—these correspond to the radial threads where the stabilimentum is located. For prey impacts at I_TR_ and I_BR_, smaller delays are observed, as vibrations first travel through radial threads adjacent to the prey location, crossing the geometric center of the web before reaching the stabilimentum threads. For prey located at I_R_, there is minimal delay, as this direction is orthogonal to the stabilimentum. In the juvenile case (J, top right panel of Figure S4), a similar trend is evident, though the overall delay is smaller (between 5 and 10 µs), due to the lower density of stabilimentum elements. This suggests that a higher density of stabilimentum elements leads to a greater increase in vibration delay for the transverse vibration direction. The reduced stabilimentum case (R, bottom left panel of Figure S4) shows results similar to the normal case (N) for prey impacts at I_B_ and I_BR_, suggesting that the effect of the stabilimentum is localized and restricted to the specific regions in which it is present. The platform stabilimentum (P, bottom central panel of Figure 3) shows a more uniform time delay, ranging from 5 to 15 µs, regardless of prey location. This is due to the roughly circular shape of the stabilimentum, which distributes additional material evenly around the web. Lastly, the drafted stabilimentum (D, bottom right panel of Figure S4) exhibits behaviour similar to the juvenile case (J) for prey impacts at I_B_ and I_BR_, reinforcing the parallel between the normal (N) and juvenile (J) stabilimenta.


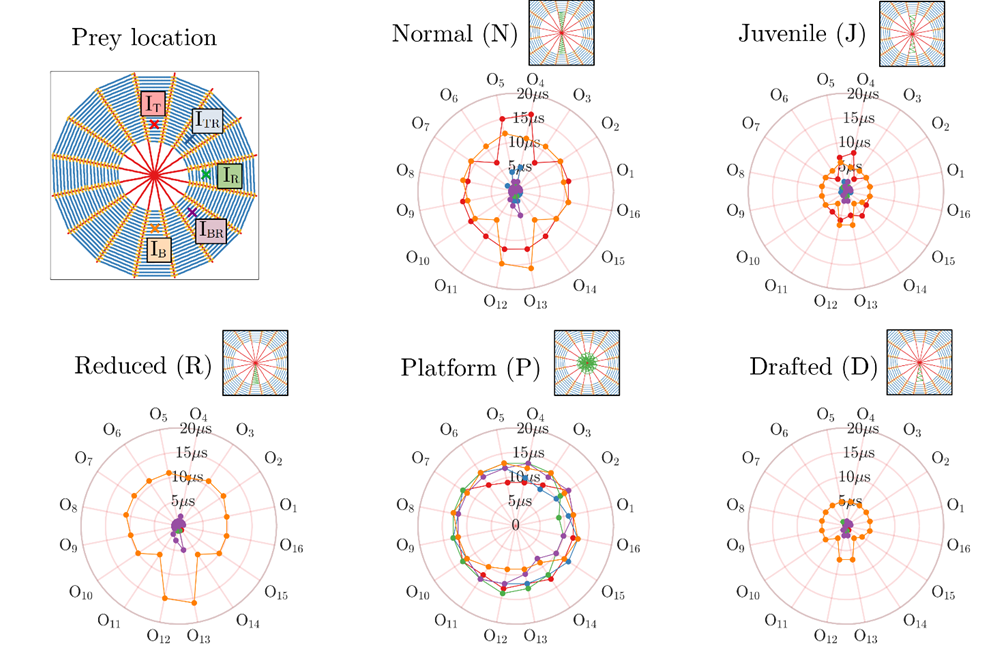


*Figure S4: Time delays (in μs) necessary for the output points located at each radial thread (O_1_-O_16_) of orb webs with stabilimentum (N, J, R, P, D) to achieve a minimum displacement threshold (3.75 μm) when compared to the baseline case (absent stabilimentum) for a prey of 0.2 mg mass. The considered prey locations (I_T_, I_TR_, I_R_, I_BR_, and I_B_, marked in red, blue, green, purple, and orange, respectively) are shown in the top left panel for reference. In the polar plots, the angular coordinates denote the radial thread output point and the radial coordinate indicates the corresponding time variation (in μs, delays are positive). The normal stabilimentum case (N, top central panel) indicates time delays of circa 15 μs for the output point pairs O_4_/O_5_ and O_12_/O_13_, respectively, for the prey location points I_T_ and I_B_. The juvenile case (J, top right panel) indicates a qualitatively similar behavior as the N case, with a smaller time delay (between 5 and 10 μs). The reduced case (R, bottom left panel) indicates a similar behavior as the N case for the impact points I_B_ and I_BR_. The platform case (P, bottom central panel) presents additional delays in all directions (between 5 and 15 μs) due to the nearly circular distribution of stabilimentum elements. The drafted case (D, bottom right panel) presents a qualitative distribution similar to the R case with a smaller time delay (circa 5 μs).*

*Time delays computed for heavier prey*

Here the results obtained for the time delays computed for the case of a mass with prey 2.0 mg are reported in Figure S5. The procedure is identical to the one used to obtain Figure S4, except for the mass of the prey.

*
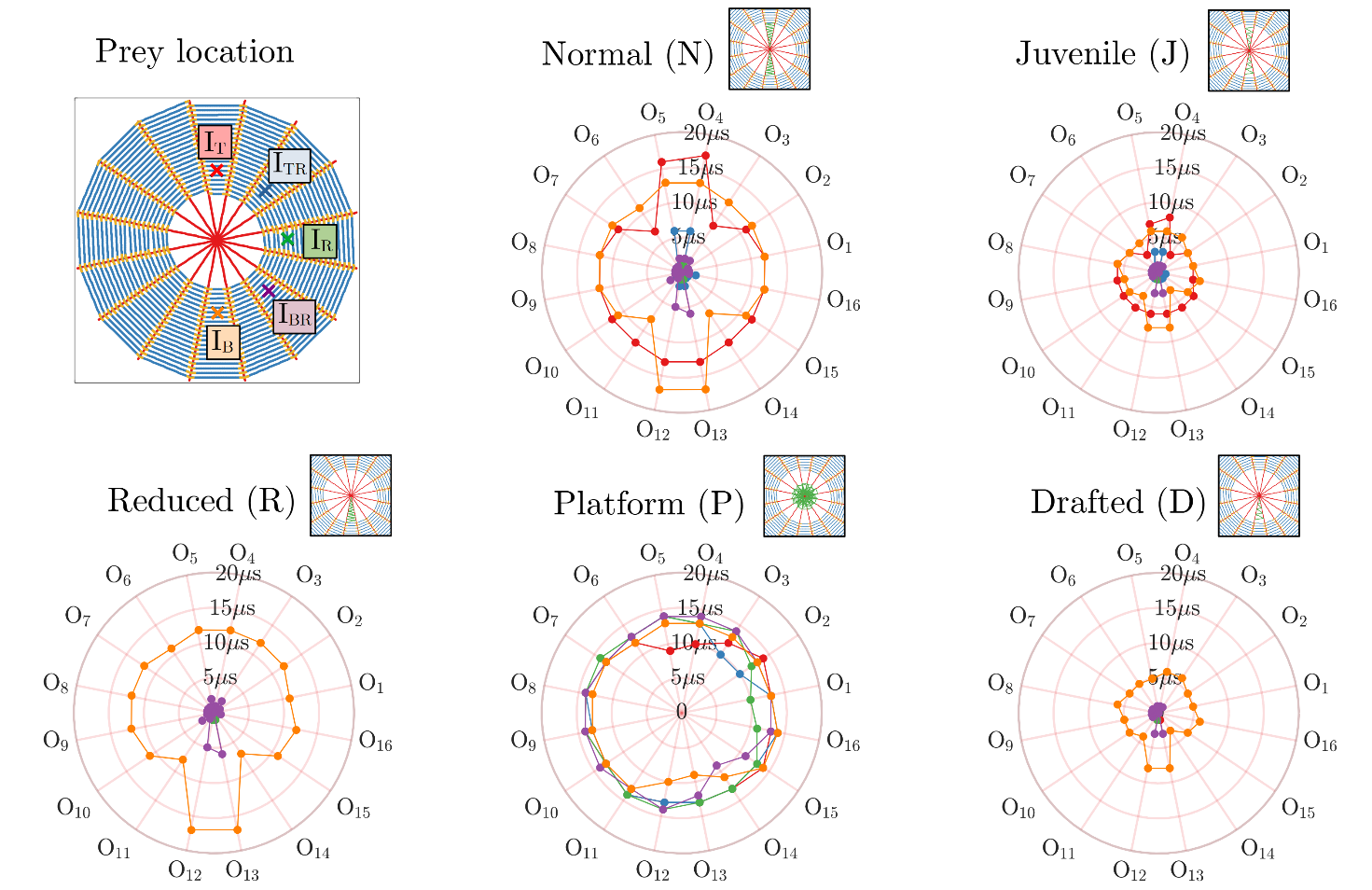
*

*Figure S5: Time delays (in μs) necessary for the output points located at each radial thread (O_1_-O_16_) of orb webs with stabilimentum (N, J, R, P, D) to achieve a minimum displacement threshold (1 μm) when compared to the baseline case (absent stabilimentum) for a prey of 2.0 mg mass. The considered prey locations (I_T_, I_TR_, I_R_, I_BR_, and I_B_ marked in red, blue, green, purple, and orange, respectively) are shown in the top left panel for reference. In the polar plots, the angular coordinates denote the radial thread output points and the radial coordinate indicates the corresponding time delays (in μs). The normal stabilimentum case (N, top central panel) indicates time delays of more than 10 μs for the output point pairs O_4_/O_5_ and O_12_/O_13_, respectively, for the prey location points I_T_ and I_B_. The juvenile case (J, top right panel) indicates a qualitatively similar behaviour as the N case, with a smaller time delay (circa 5 μs). The reduced case (R, bottom left panel) indicates a similar behavior as the N case for the impact points I_R_, I_B_, and I_BR_. The platform case (P, bottom central panel) presents additional delays in all directions (between 5 μs and 15 μs) due to the nearly circular distribution of stabilimentum elements. The drafted case (D, bottom right panel) presents a qualitative distribution similar to the R case with a smaller time delay (circa 5 μs).*

**Additional references:**

[1] Cook, R. D. (2007). Concepts and applications of finite element analysis. John wiley & sons.

[2] Bathe, K. J. (2006). Finite element procedures. Klaus-Jurgen Bathe.
