# Supplementary material for: The effect of different structural decoration geometries on vibration propagation in spider orb webs
Source: PLoS One. 2025 Oct 29;20(10):e0332593. doi: 10.1371/journal.pone.0332593 (PMC12571255; doi:10.1371/journal.pone.0332593)
Supplement: Attachment — Submitted filename: Reply to reviewers.docx [file pone.0332593.s003.docx]

**Reviewer 1:**

Orb webs of diurnal spiders often have silk attachments over or around web hub, the function of which are the subject of long-lasting controversy. This study focused the question, paying attention for the variation of form of silk decoration. The study simulated the vibrations emitted by prey caught in webs and examined the effect of different silk decoration morphologies on their propagation velocity.

The questions addressed in this study are worth investigating. The author's hypothesis that silk decoration may influence prey detection is interesting. However, I have concerns about the authors’ conclusions.

Reply:

We thank the reviewer for this valuable comment. In response, we have significantly improved the manuscript, and we hope that it is now considered suitable for publication. Notably, we have added new data and additional simulations (the new data and text were not highlighted for the sake of readability), which have led to a substantial revision of the key message of the work. We are grateful to the reviewer for providing important suggestions that contributed to this major improvement.

Simulation results showed that vibration transmission was more delayed in the web with silk decoration. I agree with that these results were opposite to the expectation of prey detection enhance hypothesis. Nevertheless, spiders may use amplitude information to detection threshold which was not examined in this study. Moreover, the authors did not examined longitudinal vibration which may also be used by spiders. Therefore, it is premature to conclude that silk decoration does not link to enhancing prey capture efficiency from the results of this study.

Standing on this, I cannot agree with the authors’ another conclusion about defensive function. There are various hypothesis for the function of silk decoration, the lack of supporting evidence for prey capture/detection hypothesis does not always indicate the support for defence hypothesis.

I recommend that the authors conduct additional analysis or that the manuscript be reorganized so that the authors only state conclusions based on their findings.

Reply:

We thank the reviewer for this valuable input. In response, we have added simulations for two additional wave propagation modes and revised the conclusions accordingly. Specifically, we now include simulations of waves propagating tangentially and normally with respect to the spiral threads.

I have other concerns. The authors use the term “stabilimentum” throughout the manuscript. However, the term comes from “to stabilize web”. Now, a lot of other functions were proposed, as mentioned in introduction, I suppose that“silk decoration” is a more appropriate term.

Reply:

We thank the reviewer for this suggestion. While it is well known that the *stabilimentum* does not contribute to the mechanical stability of the web, the term *stabilimentum* is consistently used in the literature. For the sake of clarity and consistency with established terminology, we have chosen to retain its use. To address potential confusion, we have added/modified the following lines:

Lines 20:

“The *stabilimentum*, or structural decoration, in spider orb webs is a fascinating structure.”

Lines 46-47:

“A particularly intriguing aspect of these webs is the *stabilimentum* (plural: *stabilimenta*), a fascinating structural decoration of the web.”

Lines 55-57:

“Although the word suggests otherwise, it is well established that *stabilimentum* does not improve the structural stability of the web^23^.”

In introduction, the rationale of the connection between the presence of silk decoration and vibration propagation was not fully developed. No previous studies about silk decoration is directly connected with vibration. Therefore, I recommend the authors to explain more throughly why the effect of silk decoration on vibration propagation should be examined and what outcomes would be expected in introduction.

Reply:

We thank the reviewer for this suggestion. To improve the manuscript, we have added/modified the following lines in the manuscript:

Lines 67-72:

“The presence of additional structural elements in a spider web is likely to influence its dynamic properties by modifying the web’s geometry and, consequently, altering the pathways through which specific vibrations travel^38^. In particular, the propagation of elastic waves depends not only on the material properties but also on the geometry of the structural components through which they move. Therefore, in principle, the propagation of elastic waves in webs containing a *stabilimentum* should differ from that in webs without such a structure.”

L77. I happened to be aware that the phrase “is added to neighboring radii in a zigzag manner” coincides perfectly with one appeared in Foelix’s book. Although the book is referred, I recommend the authors to rephrase it.

Reply:

We thank the reviewer for pointing this out. Indeed, we based our description of how spiders add the *stabilimentum* on Foelix’s book. We have rephrased the sentence accordingly.

Figure 1c. The scheme for Juvenile type is somewhat different from actual silk decoration appeared in the photo.

Reply:

We thank the reviewer for noticing this. We have modified the figure accordingly.

**Reviewer 2:**

I am a biologist, and have no expertise in bio-materials, and I thus cannot evaluate the major aspects of this paper relating to quantification of elastic wave propagation and the resulting perspectives on bio-inspired metamaterials. I have only two comments in this area. I wonder why the authors used only out-of-plane vibrations, when previous work (Klärner, D., and F. G. Barth. 1982. Vibratory signals and prey capture in orb-weaving spiders (Zygiella x-notata, Nephila clavipes; Araneidae). Journal of Comparative Physiology A: Neuroethology, Sensory, Neural, and Behavioral Physiology 148:445–455) concluded that, for the spider, the most important vibrations of non-sticky lines in orbs are longitudinal rather than tranverse. This gives reason to question the biological implications of this work on elastic wave propagation, but does not necessarily impact implications regarding bio-inspired metamaterials. A second comment is that the spider’s own weight, while she rests at the hub, must alter the tensions on different radii and hub lines substantially. Especially strong increases in tensions would be expected at the two sites held by the tarsi of the spider’s legs IV (increasing tensions above and decreasing just below these tarsi); such tension modifications would be expected to affect transmission of vibrations. I do not see how this effect was included in the simulations.

Reply:

We thank the reviewer for raising these two important points. We acknowledge that our initial study was limited in the types of waves analyzed. To address this, we have added simulations of two additional wave types: waves propagating tangentially to the spiral threads and normally (the new data and text were not highlighted for the sake of readability).

We also agree that the spider’s weight is an important factor in this analysis. In our study, we considered a spider weighing 20 mg, with its weight evenly distributed across the radial threads. While the magnitude of the weight itself is not expected to significantly affect the study’s outcomes, we recognize that different weight distributions could have an impact. We have acknowledged this limitation in the manuscript and are currently investigating it for a future study, as this will require an extensive analysis of spider positioning on the webs and the development of a new set of simulations.

We invite the reviewer to examine the new results regarding the additional wave types. For clarity and completeness, we have also added/modified the following lines concerning the issue of weight distribution:

Lines 318-320:

“Moreover, the spider's position on the web could play a role in mitigating/enhancing such change in prey perception time, since different positions alter spider weight distribution on the web^48^.”

On the biological side of the paper, I believe that the findings in this study are of doubtful importance for discussion of the possible functions of stabilimenta for the spiders. I would advise the authors to omit most of what they say about the functions of stabilimenta and focus their paper on elastic wave propagation. This would require substantial, but only a relatively simple modifications of the ms.

Reply:

We thank the reviewer for this comment. We have revised the manuscript to omit most of the biological speculations that are not supported by our data. Along with the additional analyses, we believe the manuscript now approaches the *stabilimentum* from a more engineering-focused perspective.

My doubts regarding the function(s) of stabilimenta stem from the following points. 1) The wide variation that the authors document in stabilimentum design (long known in this and other species) is itself an argument against the importance of its effects on elastic wave propagation; if more rapid propagation were important in promoting prey capture, then one would not expect webs with more elaborate stabilimenta (and vice versa) (the variation in stabilimentum design is explained by alternative hypotheses for stabilimentum function such as protection from visually orienting predators).

Reply:

We thank the reviewer for raising this very important point. For the sake of clarity and completeness, we have added/modified the following lines:

Lines 330-338:

“Our results contribute to a better understanding of the role *stabilimenta* play in shaping the dynamic properties of orb webs, revealing that prey detection seems to be influenced only by vibrations propagating tangentially to the spiral threads. However, from a biological standpoint, the high variability in *stabilimentum* geometry suggests that the observed differences in elastic wave propagation are unlikely to have a consistent or significant functional role. For this reason, our findings are better interpreted from an engineering perspective. They open up new possibilities for the design of bio-inspired metamaterials with tunable anisotropic wave propagation properties—for example, spiderweb-inspired lattices that allow controlled delays in wave transmission along specific pathways.”

2) The fact that many orb weaving species remove the entire central portion of the hub would require additional explanation if propagation of elastic waves in the hub has an important effect promoting prey capture.

Reply:

We agree with the reviewer on this point. However, in the present work, we focus on a simplified model of the web of *Argiope bruennichi*, as a detailed comparative study across different species would require further extensive investigation.
For the sake of completeness and clarity, we have added/modified the following lines:

Lines 344-346:

“Based on these observations, we performed numerical simulations on simplified web geometries to investigate how these decorations influence vibration propagation within the orb web.”

Lines 115-117:

“For the sake of simplicity, our models did not include the web hub—the central region of the web, which is often removed after construction by certain species, such as *Argiope bruennichi*.”

3) The first vibrations conveying the information needed by the spider at the hub regarding the location where the prey is located do not arrive from the central portions of the hub, but from beyond the hub. Propagation of elastic waves through the hub, which in Figs. 2a and 2c,d seem to suggest was the phenomenon that was modeled in this study, is not obviously related to this.

Reply:

See previous comment.

4) The constrained positions of the spider’s legs, whose mimicry of the form of the stabilimentum in many other species (including other Argiope species) is explained by alternative hypotheses of protection from predators and prey capture, but is not explained by the elastic wave propagation hypothesis.

Reply:

We thank the reviewer for this comment. In this work, we limit our analysis to the geometries of the *stabilimentum* and do not consider the position of the spider’s legs. We acknowledge this as a limitation and have noted it in lines 318-324. However, a detailed investigation of leg positioning is beyond the scope of the present study.

Minor points:
As the authors say, their conclusion from their survey that stabilimentum forms vary intra-specifically is not new. As such, it is data perhaps appropiate for a specialist spider journal, not PLoS. Perhaps they can document contrasts between their observations and previous studies.

Reply:

We thank the reviewer for this comment. While we understand that these results may also appeal to a more specialized audience in arachnology, we believe that the engineering perspective presented broadens their relevance. Therefore, we are confident that *PLOS* is an ideal platform to reach a wide and interdisciplinary audience who will benefit from these findings.

Line 36 The claim that orbs “optimize mechanical performance” in biological terms (costs of materials, construction behavior and maintenance versus payoff in materials and energy from prey captured due to vibration transmission, interception and retention of prey in nature) is not documented in the references cited (nor in any analysis of any orb web, despite frequent claims to the contrary in the literature). Various factors have been studied separately, as mentioned in the following lines in the ms; but the balance is unknown (and extremely difficult to document convincingly). The claim for optimization needs to be removed.

Reply:

We thank the reviewer for this comment. We have removed the claim.
