# Supplementary material for: The effect of different structural decoration geometries on vibration propagation in spider orb webs
Source: PLoS One. 2025 Oct 29;20(10):e0332593. doi: 10.1371/journal.pone.0332593 (PMC12571255; doi:10.1371/journal.pone.0332593)
Supplement: Attachment — Submitted filename: Reply to reviewers_rev.docx [file pone.0332593.s004.docx]

**Reviewer 1:**

In the revised manuscript, the authors had incorporated the two other types of vibration, in addition to transverse vibration, in investigating the effect of stabilimentum on the transmission of vibratory signals through spider’s orb web. I feel that the current research methodology is more consistent with the objectives of this study than the original manuscript. Nevertheless, several minor concerns remain in the organization of the manuscript, especially for the presentation of the results and significance of this study. I have listed them below.

Reply:

We thank the reviewer for this positive comment. We have modified the manuscript and we hope that it is now considered suitable for publication.

I suggest that the authors might add some sentences to explain the importance of vibration transmissiion through the web to spider’s foraging success in introduction, perhaps before explaining the effect of stabilimentum on vibration transmission, i.e., line 67. I saw such an explanation in lines 305-306, but it had better be appeared earlier. Here, the author may better introduce that some spiders are considered to control how vibration signal emitted from trapped prey travel through the web via adjusting thread tension by adding stabilimentum (Watanabe 1999) or behaviorally (Nakata 2010, 2013).

Nakata, K. (2010). Attention focusing in a sit-and-wait forager: a spider controls its prey-detection ability in different web sectors by adjusting thread tension. Proceedings of the Royal Society B: Biological Sciences, 277, 29-33. https://doi.org/10.1098/rspb.2009.1583
Nakata, K. (2013). Spatial learning affects thread tension control in orb-web spiders. Biology Letters, 9(4). https://doi.org/10.1098/rsbl.2013.0052

Reply:

We thank the reviewer for this valuable input. We have added the suggested discussion and the suggested references.

Lines 91-92: In Figure 1a, the spider seems to wrap a prey with silk, not spin stabilimentum. Although it’s true that spiders use the same type or silk for wrapping prey and stabilimentum, I am afraid that the text here is misleading. Additionally, the authors may refer a recent literature by Lee and Moon (2025).

Lee, S. M., & Moon, M. J. (2025). Structural and Functional Analyses of Stabilimentum in the Garden Spider, Argiope bruennichi (Araneae: Araneidae). Entomological Research, 55(2), e70023.

Reply:

We thank the reviewer for this suggestion. We have improved the text to avoid confusion and added the suggested reference.

Lines 122-126: In the literature studying vibration transmission through webs, these three types of vibrations are often referred to as longitudinal, transverse, and lateral vibrations. For consistency, I suggest that authors follow this nomenclature or at least explain the relationship between the terms used in this study and those used in the previous literatures.

Reply:

We thank the reviewer for this suggestion. We have now explained the relationship among the terms, for the term lateral might sound odd from the engineering point of view.

Lines 202-203: In Figure 3, the lines for each type of stabilimentum could not be seen, except for line A. Did this happen because all the lines appear to overlap with little difference between them? If so, it should be better explained in Figure legend. I had similar quesions and comments for Figure 4 and 5.

Reply:

We thank the reviewer for pointing this out. Indeed, the lines overlap. For this reason, we have modified the text accordingly.

Lines 228-239: It was not easy for me to understand that whether stabilimentum decreased time necessary to achieve threshold displacement. Line 228 read there was a mild effect, but line 237 read there was no significant effect. I understood these sentences were not contradictory with each other only after reading lines 242-243. If the authors considered that the decrease in time caused by N stabilimentum when the prey location is orthogonal to the direction of stabilimentum was exceptional case, consider the order of presenting results accordingly.

Reply:

We thank the reviewer for this comment. We have now modified the text to make it clearer, and referred to the specific cases (e.g., N or A types of stabilimentum).

**Reviewer 3:**

The paper is interesting and topical. The idea is original and the analyses seem to be well implemented.
I can see the paper has had a review already so I see that the previous reviewers provided detailed feedback that the author has now implemented.
I do want to pick up on some issues and/or erroneous pieces of information presented. I expect the authors to address these in order to complete this nice work.

Reply:

We thank the reviewer for his useful comments. We have improved the manuscript accordingly.

Stabilimentum are sometimes just called web decorations, as per the explanations in Herberstein et al. 2000. Please explain this as it helps readers find relevant literature.

Stabilimentum does not need to be italicized.

Reply:

We thank the reviewer for this comment. We have revised the manuscript to explain the terminology and remove italic font for the word stabilimentum.

Much of the literature presented herein is somewhat outdated. I recommend the authors refer to the paper by Yeh et al. 2015 in Scientific Reports on the simultaneous top down (from predators) and bottom up (from prey) pressures acting on stabilimentum form selection by individuals. The important point made here is about prey (primarily honeybees) learning to distinguish certain stabilimentum designs and avoiding webs, and predators (primarily wasps) learning to recognize and use them to hunt spiders.

Reply:

We thank the reviewer for this input. We have now added the suggested reference alongside the ones suggested by reviewer 1.

Please add some lines about this work (as well as that by Cheng et al. 2010 in J. Exp Biol. on insect form vision implications) within the Introduction. It will also assist you to more strongly argue a case for their being large natural variations in stabilmenta use, to which you can argu there is a neglected cost – that of reduced vibratory propagation (thus reduced mating and feeding opportunities).

Reply:

We thank the reviewer for the suggestion. We have inserted a sentence related to the paper in the introduction.

Line 62-63: Stabilimenta absolutely do emit a stronger UV signal than other spider silk. See Blamires et al. 2019 (J Roy Soc Interface) and 2020 (Roy Soc Open Sci) for spectra measured on Major Ampullate silks for instance. The spectra show a deep depression in the UV range.

Reply:

We thank the reviewer for suggesting these interesting works, which have now been implemented in the Introduction.

Line 65: protection against predators is certainly not the most well supported hypothesis for stabilimentum use by Argiope spp. (it is for Cyclosa spp. nonetheless). Prey luring is by far more widely shown. Again refer to Yeh et al. and the explanation therein as to how the 2 forces shape stabilimentum use.

Reply:

We thank the reviewer for this comment. The reference has been added following the previous comments, and here we have now modified the text to be less conclusive in our statement.

Give more detail in your methods about how you controlled variables and ran the simulations as well as the actual web stabilimenta variations considered.
There are form variations not represented in Figure 1. Explain these and how including them in a model might affect the outcomes.

Reply:

We thank the reviewer for these comments. We have added two sentences in the Methods section to provide additional details on the simulations and on how different geometries may influence them. We would like to emphasize that these simulations should be interpreted qualitatively rather than quantitatively, as several parameters can significantly affect the outcomes. In the Supplementary Information, we have already highlighted some of the most relevant factors, such as prey body mass and the distribution of the spider’s weight on the radial threads.
